# *In vitro* Radiopharmaceutical Evidence for MCHR1 Binding Sites in Murine Brown Adipocytes

**DOI:** 10.3389/fendo.2019.00324

**Published:** 2019-06-11

**Authors:** Theresa Balber, Katarína Benčurová, Florian Wolfgang Kiefer, Oana Cristina Kulterer, Eva-Maria Klebermass, Gerda Egger, Loan Tran, Karl-Heinz Wagner, Helmut Viernstein, Katharina Pallitsch, Helmut Spreitzer, Marcus Hacker, Wolfgang Wadsak, Markus Mitterhauser, Cécile Philippe

**Affiliations:** ^1^Division of Nuclear Medicine, Biomedical Imaging and Image-Guided Therapy, Medical University of Vienna, Vienna, Austria; ^2^Department of Pharmaceutical Technology and Biopharmaceutics, Faculty of Life Sciences, University of Vienna, Vienna, Austria; ^3^Department of Nutritional Sciences, Faculty of Life Sciences, University of Vienna, Vienna, Austria; ^4^Division of Endocrinology and Metabolism, Department of Medicine III, Medical University of Vienna, Vienna, Austria; ^5^Ludwig Boltzmann Institute Applied Diagnostics, Vienna, Austria; ^6^Department of Pathology, Medical University of Vienna, Vienna, Austria; ^7^Institute of Organic Chemistry, University of Vienna, Vienna, Austria; ^8^Department of Pharmaceutical Chemistry, Faculty of Life Sciences, University of Vienna, Vienna, Austria; ^9^Center for Biomarker Research in Medicine – CBmed, GmbH, Graz, Austria

**Keywords:** melanin-concentrating hormone receptor 1, MCHR1, brown adipocytes, BAT, adrenergic beta-3 receptor, [^18^F]FE@SNAP, [^11^C]SNAP-7941, PET

## Abstract

[^11^C]SNAP-7941 and its radiofluorinated, fluoro-ethyl derivative [^18^F]FE@SNAP have been developed as the first positron emission tomography tracers for melanin-concentrating hormone receptor 1 (MCHR1) imaging. Accumulation of these MCHR1 PET-tracers in rat brown adipose tissue (BAT) *in vivo* provided first indication of MCHR1 expression in rodent BAT. To rule out off-target binding, affinity of both MCHR1 ligands toward adrenergic beta-3 receptors (ADRB3) was examined. Further, specific binding of [^11^C]SNAP-7941 to brown adipocytes and effects of MCHR1 ligands on brown adipocyte activation were investigated. SNAP-7941 and FE@SNAP evinced to be highly selective toward MCHR1. [^11^C]SNAP-7941 binding to brown adipocytes was shown to be mainly MCHR1-specific. This data strongly indicates MCHR1 expression in rodent BAT and moreover, a peripheral, anti-obesity effect of MCHR1 antagonists directly exerted in BAT is proposed. Moreover, MCHR1 expression in murine brown adipocytes was confirmed by protein and mRNA analysis. We conclude that MCHR1 PET imaging contributes to basic research in endocrinology by elucidating the involvement of the MCH system in peripheral tissues, such as BAT.

## Introduction

Although the physiological relevance of brown adipose tissue (BAT) was underestimated for a long time, it nowadays attracts scientific interest as a therapeutic target for obesity and related metabolic diseases such as type 2 diabetes (1–3). The groundbreaking discovery of functional BAT depots in adult humans contributed significantly to the re-introduction of BAT in metabolic research and was enabled by positron emission tomography (PET) using the glucose analog 2-[^18^F]fluoro-2-deoxy-D-glucose ([^18^F]FDG) combined with computer tomography (CT). The fact that BAT activity, quantified as [^18^F]FDG uptake, was negatively correlated to body mass index and percent body fat indicated a potential role of BAT in energy expenditure in humans (4–6). PET is a sensitive and non-invasive *in vivo* imaging technique that directly visualizes different molecular interactions or metabolic processes depending on the used radiolabeled tracer in a target tissue. Thus, besides [^18^F]FDG, also other PET-tracers were investigated to study BAT function, namely [^11^C]meta-hydroxyephedrine, [^11^C]acetate, 14-[^18^F]fluoro-6-thiaheptadecanoic acid, and (S,S)-*O*-[methyl-^11^C] methylreboxetine thereby enhancing the understanding of BAT activation, control, and metabolism (7–9).

Evolutionary, BAT functions as an energy dissipating organ by exerting non-shivering thermogenesis and thus maintaining body temperature during cold exposure. This heat-producing process is controlled by the sympathetic nervous system leading to noradrenaline release followed by activation of adrenergic beta-3 receptors (ADRB3) in brown adipocytes. Subsequent lipolysis or increased uptake of glucose and its conversion into fatty acids finally lead to the uncoupling of ATP synthesis *via* uncoupling protein 1 (UCP-1) resulting in increased fatty acid oxidation and dissipation of excess energy as heat. Thereby, BAT regulates fuel metabolism by increasing glucose and triglyceride uptake, decreasing blood glucose and lipids, and by serving as glycogen storage (1, 10–14).

Obesity results from a chronic energy imbalance, when food intake exceeds total body energy expenditure (15). The melanin-concentrating hormone, a neuropeptide predominantly expressed in the lateral hypothalamus and zona incerta, is involved in the control of appetite and food intake (16, 17). In fact, upregulated MCH expression was found in the hypothalamus of obese and leptin-deficient mice and is moreover induced by fasting in wild-type mice (16). Accordingly, MCH-deficient mice are lean due to hypophagia and have an increased metabolic rate (18). Besides, MCH was shown to stimulate leptin secretion in rat white adipocytes and MCH was detected in rat plasma (19). In rodents, MCH exerts its effects solely by stimulation of the melanin-concentrating hormone receptor 1 (MCHR1), as rodents do not express melanin-concentrating hormone receptor 2 (MCHR2). Several centrally active MCHR1 antagonists have been developed for the treatment of obesity (20). In a diet-induced obesity mouse model it was shown that the anti-obesity effects of the tested MCHR1 antagonist are not only due to suppression of feeding, but also to a stimulation of energy expenditure. A significantly increased body temperature in MCHR1 antagonist-treated mice suggested a potential involvement of the MCH system in the regulation of energy expenditure *via* BAT (21). It was reported that a large proportion of neurons in the lateral hypothalamus projecting to BAT contain MCH (22). Thus, a central effect of the MCHR1 antagonist and subsequent transmission to BAT was presumed, as a direct effect on BAT could not be shown (21).

[^11^C]SNAP-7941 and its fluoro-ethylated analog [^18^F]FE@SNAP—both MCHR1 antagonists—have been developed as the first PET-tracers for MCHR1 imaging in our group (23–28). Recent μPET experiments in naïve rats showed uptake of [^18^F]FE@SNAP and [^11^C]SNAP-7941 in BAT, though MCHR1 expression has previously not been reported in this tissue. Surprisingly, administration of a pharmacological dose (15 mg/kg BW) of unlabeled SNAP-7941 for displacement purposes caused uptake enhancement of both MCHR1 PET-tracers in interscapular BAT depots (29). These observations suggest activation of BAT by MCHR1 antagonists. However, they were contradictory to earlier performed biodistribution experiments in conscious rats, when animals received a pharmacological dose of SNAP-7941 (15 mg/kg BW) or vehicle 30 min prior to [^18^F]FE@SNAP application *via* a jugular vein catheter. *Ex vivo* autoradiography and *ex vivo* biodistribution demonstrated significant blocking of [^18^F]FE@SNAP uptake in BAT of conscious rats, indicating specific [^18^F]FE@SNAP binding (30). The discrepancy in anesthetized and conscious rats suggests a potential influence of the applied anesthesia on μPET acquisition.

Based on these *in vivo* findings, MCHR1-selectivity of FE@SNAP and SNAP-7941 has to be proven to avoid misleading interpretation of PET imaging data. To evade molecular alterations caused by anesthesia, we decided in favor of an *in vitro* approach. Therefore, in this preclinical *in vitro* study, affinity of both ligands toward the ADRB3, which is the receptor predominantly involved in BAT activation, was determined. Moreover, the potential involvement of the MCHR1 in BAT was investigated using brown adipocytes and the respective PET-tracer [^11^C]SNAP-7941 and additionally, [^18^F]FDG as a surrogate marker for brown adipocyte activity. Within the scope of this *in vitro* study, we aimed at applying conventional binding assays and kinetic radioligand binding measurements to cover binding interactions with single receptor sites as well as whole cell binding studies.

## Methods

### General

2-[^18^F]fluoro-2-deoxy-D-glucose ([^18^F]FDG) was prepared in-house using a fully automated cassette-based synthesizer (FASTlab, GE Healthcare, Uppsala, Sweden) within the clinical routine production at the Vienna General Hospital, Austria. [^11^C]SNAP-7941 was synthesized as reported elsewhere using an automated module (TRACERlab FC X Pro, GE Healthcare, Uppsala, Sweden) (25). All PET-tracers were physiologically formulated and quality-controlled prior to administration. The adrenergic receptor beta ligands carazolol, pindolol and (S)-propranolol hydrochloride were purchased from Sigma-Aldrich (St. Louis, USA). The ADRB3 agonist CL 316,243 was purchased from Tocris Bioscience (Bristol, UK). The unlabeled compounds FE@SNAP and SNAP-7941 were synthesized at the Department of Pharmaceutical Chemistry and at the Department of Organic Chemistry (University of Vienna, Austria). The radioligands 5,7-[^3^H](-)CGP-12177 and [^125^I](-)Iodocyanopindolol were purchased from PerkinElmer, Inc. (Waltham, USA). All other reagents and cell culture supplies were purchased from standard commercial sources.

### Cell Culture

Cells were maintained under standard conditions in a humidified incubator (37°C, 5% CO_2_). CHO-K1 cells expressing the human adrenergic beta-3 receptor (CHO-K1-ADRB3) were purchased from PerkinElmer (ValiScreen® ES-035-C). CHO-K1-ADRB3 were grown using Ham's F12 (Nut Mix) supplemented with 10% FCS, 2 mM L-glutamine, penicillin (100 Units/mL), streptomycin (100 μg/mL), and 0.4 mg/mL geneticin. CHO-K1 cells were a generous gift from Prof. Karl Norbert Klotz (University Würzburg, Germany) and cultured likewise without selection antibiotics. Immortalized murine brown pre-adipocytes were provided by the Department of Medicine III (Medical University of Vienna, Austria) and cultured in high glucose Dulbecco's Modified Eagle's Medium (DMEM) supplemented with 10% FBS, 1 mM pyruvate, penicillin (100 Units/mL) and streptomycin (100 μg/mL). When cells were confluent, differentiation to brown adipocytes was induced for 48 h using 1.72 μM insulin (human recombinant), 1 nM thyroid hormone (T3), 0.5 mM 3-isobutyl-1-methylxanthine (IBMX), 125 nM indomethacin, and 1 μM dexamethasone. After induction, the medium was changed to post-differentiation medium (high glucose DMEM) supplemented with 10% FBS, 1 mM pyruvate, penicillin (100 Units/mL), streptomycin (100 μg/mL), 1.72 μM insulin, and 1 nM T3. The post-differentiation medium was replenished every second day. Brown adipocytes were used for experimental investigations 4–6 days after induction. Oil Red O staining was performed according to the manufacturer's instructions. Counterstaining was performed using haematoxylin.

### Cell Membrane Preparation

CHO-K1-ADRB3 cell membranes were prepared from 175 cm^2^ cell culture flasks, when 80% confluence was reached. All procedures were performed at 4°C. Cells were scraped off using ice-cold lysis buffer (10 mM Tris-HCl pH 7.4, 1 mM EDTA) and protease inhibitor cocktail was added according to the manufacturer's instructions (Sigma-Aldrich, St. Louis, USA). The cell suspension was homogenized using cannulas (29 G) and centrifuged for 10 min at 1,000 × g. The obtained supernatant was centrifuged for 30 min at 100,000 × g. Subsequently, the pellet was suspended using 50 mM Tris-HCl pH 7.4 and aliquoted. Aliquots were shock-frozen in liquid nitrogen and stored at −80°C until usage. Protein concentration of the cell membrane suspension was determined using BCA Protein Assay Kit (Thermo Scientific, Waltham, USA).

### Competitive Binding Studies: Affinity Determination

All stated concentrations refer to the final assay volume of 500 μL buffer (25 mM HEPES pH 7.4, 1 mM EDTA, 0.5% BSA). Competitive binding assays were performed using 13.6 ng/μL ADRB3 expressing CHO-K1 cell membrane suspension (cf. 2.3) and 5 nM [^3^H]CGP-12177 as the respective radioligand. Test compounds [carazolol, pindolol, (S)-propranolol, FE@SNAP, SNAP-7941] were added in rising concentrations, whereby the concentration of solvent [ethanol or dimethyl sulfoxide (DMSO)] in the final assay volume remained always 1%. Nonspecific binding was determined using 10 μM carazolol. Total binding was determined in the presence of 1% respective solvent. Incubation was performed for 90 min at 27°C. Filtration through GF/B filters (Whatman®, presoaked in 0.05% PEI) was performed using a cell harvester (Brandel®, Gaithersburg, MD, USA) and was followed by two washing steps using ice-cold wash buffer (10 mM HEPES, 500 mM NaCl, pH 7.4). Filter pieces containing receptor-bound radioactivity were shaken for 30 min in scintillation cocktail (Ultima Gold™, PerkinElmer, Waltham, USA) before liquid scintillation counting (Hidex 300 SL, Turku, Finland). IC_50_ fitted binding curves were generated using the GraphPad Software 5.0 (La Jolla, CA, USA) using nonlinear regression. K_i_ values were calculated using the Cheng and Prusoff equation assuming a K_d_ value of 109.2 nM for [^3^H]CGP-12177 according to the literature (31).

### Kinetic Measurements: Displacement of [^125^I]Iodocyanopindolol Binding

2.5 × 10^5^ cells (CHO-K1, CHO-K1-ADRB3) per cell culture dish were seeded 2 days prior to the experiment and incubated in an inclined position to allow an adherent cell pole at one side of the cell dish. Cell dish preparation was performed as previously described (32, 33). Real-time kinetic radioligand binding measurements were performed using a dedicated device for low energy gamma radiation (LigandTracer® Gray Technology, Ridgeview, Sweden). The assay protocol compromised consecutive radioactivity measurements of the target region (cell pole) and of the opposite pole of the petri dish, where no cells were seeded (background signal). Radioactivity was counted in each region for 3 s with a delay of 2 s over the time course of the experiment. Raw counts per second (cps) of the target region were corrected for background signal and for radioactive decay. Displacement of [^125^I]Iodocyanopindolol binding was conducted after 1 h association phase of radioligand binding by consecutively applying rising concentrations of SNAP-7941 and FE@SNAP. The same volume of ethanol was added as vehicle control. Experiments were performed on at least three different days.

### Kinetic Measurements: Glycolytic Activity

Kinetic measurements of [^18^F]FDG uptake were performed in pre-adipocytes and differentiated brown adipocytes to investigate metabolic activity. Cell seeding and the assay protocol followed a standard procedure described above (cf. 2.5). One hour prior to the experiment, post-differentiation medium was discarded and unsupplemented, glucose-free DMEM was added, followed by incubation with [^18^F]FDG (150 kBq per dish) for another 50 min. As glucose concentration is high in cell culture media thus interfering with [^18^F]FDG uptake, cells were starved to achieve reasonable signal. Experiments were performed on at least three different days.

### [^18^F]FDG Uptake Experiments: Effects of MCHR1 Ligands on Brown Adipocyte Activation

Brown pre-adipocytes were seeded, grown to confluence and differentiated in 6-well plates as described in section General. Cell starving was performed for 1 h using unsupplemented, glucose-free DMEM. Thirty minutes prior to [^18^F]FDG incubation, cells were pre-treated with the adrenergic beta-3 agonist (2 μM CL 316,243), adrenergic beta antagonist (2 μM (S)-propranolol), the MCHR1 ligands (20 μM SNAP-7941 or FE@SNAP), or vehicle (baseline). [^18^F]FDG uptake was performed for 50 min at 37°C (humidified atmosphere, 5% CO_2_). Supernatant was taken off, cells were washed with ice-cold PBS and finally trypsinized. Radioactive cell fractions were gamma counted (2480 Wizard^2^, PerkinElmer) and normalized to percentage uptake per well. [^18^F]FDG uptake under baseline conditions refers to 100% uptake. Three independent experiments were performed in triplicates. Statistical analysis was performed using an unpaired, two-tailed *t*-test.

### Investigation of Specific [^11^C]SNAP-7941 Binding to Brown Adipocytes

Brown pre-adipocytes were seeded, grown to confluence and differentiated in six-well plates as described above. Thirty minutes prior to the experiment, maintenance medium (cf. 2.1) was discarded and replaced with respective serum- and additive-free medium to avoid plasma protein binding of the PET-tracer. Cell starving, as performed for [^18^F]FDG uptake measurements, was not indicated for [^11^C]SNAP-7941 binding experiments. Cells were pre-treated for 30 min with ADRB3 ligands (CL 316,243, (S)-propranolol, 2 μM each) or MCHR1 ligands (SNAP-7941, FE@SNAP, 2 μM each). Baseline was determined using DMSO or ethanol, respectively. Sample processing, data analysis and statistical testing were performed as described in [^18^F]FDG Uptake Experiments: Effects of MCHR1 Ligands on Brown Adipocyte Activation.

### MCHR1 Expression in Murine Brown Adipocytes

Two 6 weeks old male BALB/c mice were sacrificed and organs (brain, lung, and spleen) were removed to serve as reference tissues in Western Blot and real-time quantitative PCR (qPCR) experiments. Organs were divided and tissue samples were subjected to protein extraction and RNA extraction. Murine brown adipocytes were treated as described above (cf. 2.2) and both protein and RNA were extracted from identical subcultures on day 5 and 6 after induction.

RNA extraction of murine brown adipocytes and reference tissues was performed with TRIzol reagent (ambion®). In the case of the adipocytes, an additional centrifugation step ensured for the separation of the fatty layer. RNA concentration and purity was measured using NanoDrop (Thermo Fisher Scientific). Reverse transcription was carried out with the qScript® cDNA Synthesis Kit (Quantabio). Subsequently, two-step real-time qPCR was run on CFX96™ Real-Time System (Bio-Rad). All reactions were performed in duplicates, using the Luna® Universal qPCR Kit (BioLabs). ß*-actin* or *Rpl27* were used as endogenous controls. Sequences of the primers (eurofins) are noted in the Supplementary Material. Data was analyzed with the corresponding CFX Manager 3.1 (Bio-Rad) and Excel 2013 (Microsoft® Office). Mean Ct values of all four qPCR runs were generated and relative mRNA expression was calculated with the formula log (2^ΔΔCt^), compared to negative control (spleen).

Protein extraction was performed using commercially available radioimmunoprecipitation assay (RIPA) buffer. Protease inhibitor cocktail was added according to the manufacturer's instructions. Cell and tissue lysates were centrifuged until a clear (lipid-free) supernatant was obtained (12,000 rpm, 4°C). Protein concentration was determined using a bicinchoninic acid kit (BCA kit, Thermo Scientific) and 20 μg protein were loaded each. After gel electrophoreses (TGX™ precast gels, Bio-Rad, Laboratories, Inc.) and subsequent semi-dry blotting, Ponceau S staining was performed to ensure equal protein loading and transfer. Nitrocellulose membranes (Amersham™ Protran™, GE Healthcare Life Science) were incubated with 5% dry milk powder for 90 min. Primary antibody incubation (rabbit polyclonal anti-MCHR1, PA5-50705, Thermo Fisher Scientific, 1:1,000, expected size ~55 kDa) was performed overnight at 4°C. Membranes were further incubated with goat anti-rabbit IgG HRP conjugate (A16104, Thermo Fisher Scientific, 1:2,500, 1 h, RT), followed by chemoluminescence imaging using a substrate detection kit and a dedicated device (Bio-Rad ChemiDoc™ Imaging System). The Western Blot was normalized to Ponceau S staining using pixel analysis with the open source image processing program ImageJ. For relative quantification the formula Pixel density (bands/controls) was applied.

## Results

### Competitive Binding Studies: Affinity Determination

The two MCHR1 ligands displayed intermediate affinity toward ADRB3, as K_i_ values in μM-range were obtained (SNAP-7941: 14.5 ± 0.3 μM and FE@SNAP: 65.1 ± 2.9 μM, *n* = 3 in triplicates). However, both ligands demonstrated high selectivity toward MCHR1. For comparability, the assay was also performed using the ADRB3 ligands carazolol, pindolol and (S)-propranolol as standard compounds (Table 1).

**Table 1:** K_i_ values are given in nM or μM, respectively and were obtained from three independent experiments performed in triplicates.

**Compound**	**ADRB3 experimental**	**ADRB3 reference**	**MCHR1**	**ADRB3/ MCHR1**
carazolol	2.0 ± 0.3 nM	2.0 nM	n.d.	n.d.
pindolol	44.5 ± 11.8 nM	44.1 and 11 nM	n.d.	n.d.
(S)-propranolol	67.4 ± 14.4 nM	186 and 145 nM	n.d.	n.d.
SNAP-7941	14.5 ± 0.3 μM	n.a.	3.91 ± 0.74 nM	~3,708
FE@SNAP	65.1 ± 2.9 μM	n.a.	9.98 ± 1.12 nM	~6,523

*Reference values for standard compounds were taken from literature (43, 45–47). K_i_ values for SNAP-7941 and FE@SNAP toward MCHR1 were previously described (28). (n.a., not available; n.d., not determined)*.

### Kinetic Measurements: Displacement of [^125^I]Iodocyanopindolol Binding

**Figure 1:**
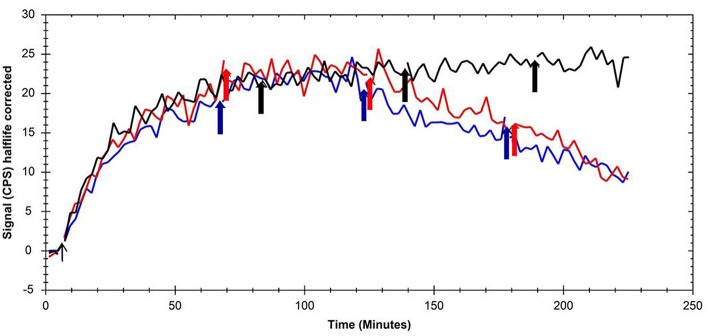
Overlay of kinetic binding toward CHO-K1-ADRB3 and displacement curves of [^125^I]Iodocyanopindolol using the MCHR1 ligands SNAP-7941 (red line) and FE@SNAP (blue line) and ethanol (black line) as the respective vehicle control. Arrows indicate the addition time points of the MCHR1 ligands and vehicle, respectively.

Additionally to the conventional affinity determination using cell membrane preparations, displacement experiments using [^125^I]Iodocyanopindolol as the ADRB3 model ligand were performed. No binding kinetics was observed for parental CHO-K1 cells (data not shown). Application of 20 μM non-radioactive compound (either SNAP-7941 or FE@SNAP) after 60 min [^125^I]Iodocyanopindolol association phase did not affect binding to CHO-K1-ADRB3 cells. A second application of 20 μM MCHR1 ligand after 120 min led to a decrease in cell-associated radioactive signal, which was more pronounced after a third addition of unlabeled compound (Figure 1). Thus, for displacement of [^125^I]Iodocyanopindolol binding, excess of unlabeled MCHR1 ligand was required pointing at the intermediate affinity of SNAP-7941 and FE@SNAP toward ADRB3. Vehicle control (ethanol) showed no effect on [^125^I]Iodocyanopindolol binding to CHO-K1-ADRB3 cells.

### Histological Staining and Metabolic Activity of Brown Adipocytes

**Figure 2:**
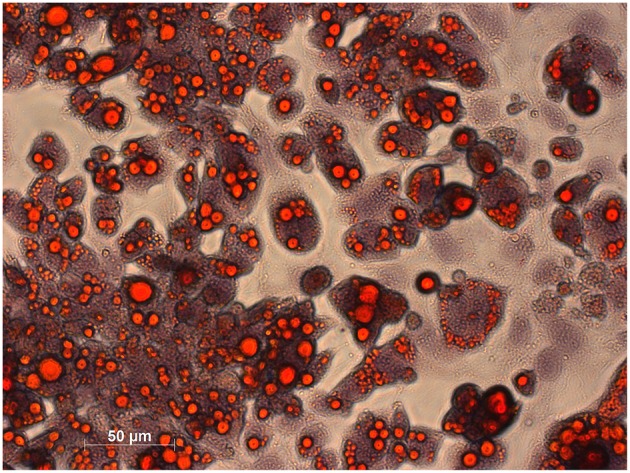
Histological staining of lipids in mature brown adipocytes was performed using Oil Red O and haematoxylin for counterstaining. Lipids appear in red and chromatin in bluish.

**Figure 3:**
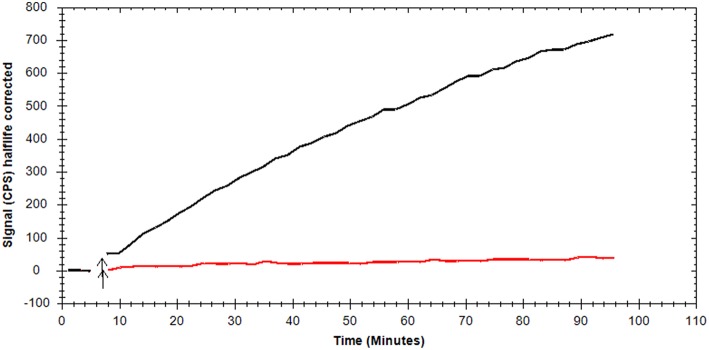
Representative, background-corrected time-activity curves of [^18^F]FDG uptake by mature brown adipocytes (black) and pre-adipocytes (red) are shown as overlay.

Morphological characterization of brown adipocytes was performed *via* Oil Red O staining. Mature brown adipocytes were obtained 4–6 days after induction. Fully differentiated brown adipocytes showed typical morphologic characteristics (spherical shape, multilocular lipid vesicles, central nucleus, Figure 2) and were thus visually distinguishable from pre-adipocytes (fibroblast-like shape). Glycolytic activity of brown adipocytes was demonstrated by means of kinetic [^18^F]FDG uptake measurements. [^18^F]FDG uptake by pre-adipocytes was negligible (Figure 3).

### [^18^F]FDG Uptake Experiments: Effects of MCHR1 Ligands on Brown Adipocyte Activation

**Figure 4:**
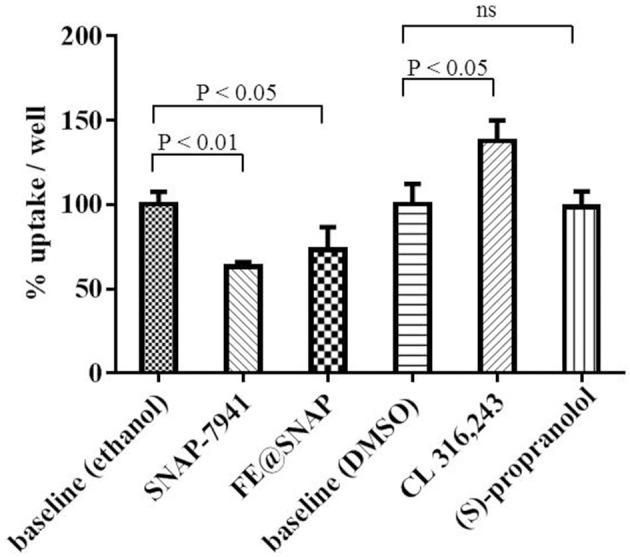
Baseline refers to 60 min [^18^F]FDG uptake in the presence of vehicle control and was normalized to 100% uptake/well. Data is presented as mean ± standard deviation (SD) from at least three independent experiments.

Pharmacological effects of MCHR1 antagonists and ADRB3 ligands on brown adipocyte activation were measured using [^18^F]FDG as a surrogate marker. The ADRB3 agonist CL 316,243 significantly (*P* < 0.05) enhanced [^18^F]FDG uptake to 137.7 ± 12.2% referring to baseline, whereas the non-selective adrenergic beta receptor antagonist (S)-propranolol did not alter [^18^F]FDG uptake. The opposite effect was observed for MCHR1 ligands, as [^18^F]FDG uptake was significantly reduced, when SNAP-7941 (63.24 ± 2.6%) or FE@SNAP (73.03 ± 13.6%) were applied (Figure 4).

### Investigation of Specific [^11^C]SNAP-7941 Binding to Brown Adipocytes

**Figure 5:**
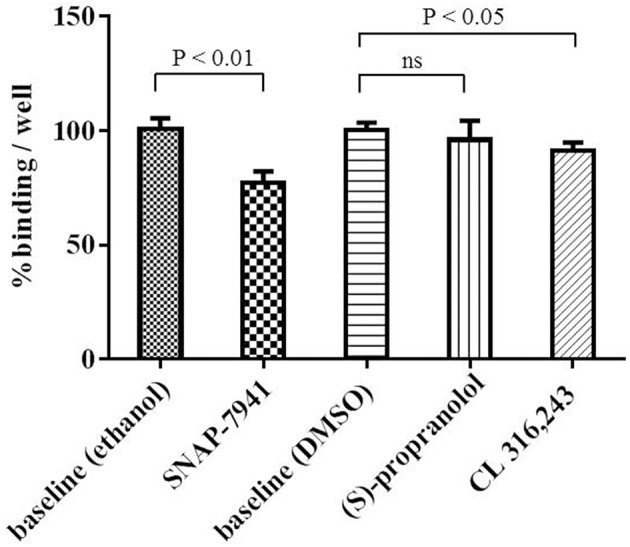
[^11^C]SNAP-7941 binding to brown adipocytes in the presence of vehicle control (ethanol or DMSO) represents baseline (100% binding/well). SNAP-7941 (2 μM) significantly reduced [^11^C]SNAP-7941 binding by 22.9 ± 5.0%. The effect of the ADRB3 agonist CL 316,243 was less pronounced and the non-selective adrenergic receptor beta antagonist propranolol did not alter [^11^C]SNAP-7941 binding. Data is presented as mean ± standard deviation (SD) from at least three independent experiments.

Competitive binding experiments were performed to investigate specificity of [^11^C]SNAP-7941 binding to brown adipocytes. Application of 2 μM unlabeled SNAP-7941 significantly reduced cell-associated radioactive signal of [^11^C]SNAP-7941 to 77.1 ± 5.0% binding per well referring to baseline (*P* < 0.01). Moreover, competition of [^11^C]SNAP-7941 using ADRB3 ligands resulted in 95.8 ± 8.6% binding/well for (S)-propranolol (not significant) and 91.1 ± 3.6% binding/well for CL 316,243, respectively (Figure 5).

### MCHR1 Expression in Murine Brown Adipocytes

**Figure 6:**
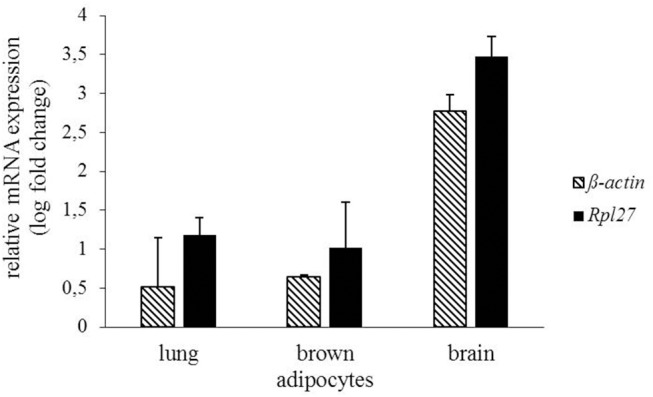
Gene expression of *Mchr1* in mouse lung, brain and murine brown adipocytes normalized to the respective housekeeping gene (*Rpl27 n* = 2, ß*-actin n* = 2). Values were calculated with respect to mouse spleen as negative control using the ΔΔCt method.

**Figure 7:**
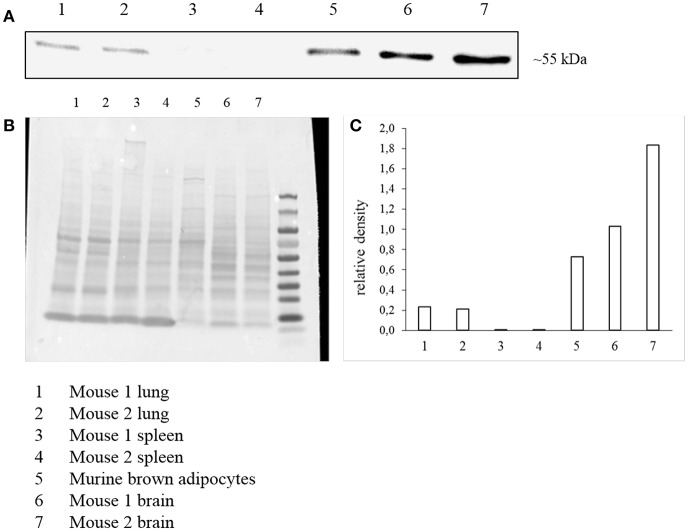
Western Blot analysis of MCHR1 expression **(A)** in murine brown adipocyte lysates and reference tissues. Data is presented as cropped image format. Corresponding Ponceau S staining as loading control **(B)** and calculated relative density of MCHR1 bands normalized to Ponceau S **(C)**.

In addition to radioligand binding, *Mchr1* mRNA and MCHR1 protein were determined in murine brown adipocytes. Highest *Mchr1* mRNA levels were found in mouse brain (positive control, mean Ct 26.4), whereas low expression was detected in mouse lung (mean Ct 35.5) and murine brown adipocytes (mean Ct 33.1). No *Mchr1* mRNA expression was found in mouse spleen (mean Ct 38.5) that served as a negative control (Figure 6). A representative figure of the melt curves is included in the Supplementary Material. In line with this, MCHR1 protein was detected in mouse brain, lung and murine brown adipocytes. No MCHR1 protein expression was found in mouse spleen (Figure 7).

## Discussion

For affinity determination toward ADRB3, a competitive binding assay using CHO-K1-ADRB3 cell membranes was established and tested using known ADRB3 ligands. The obtained K_i_ values for (S)-propranolol and pindolol differed slightly from literature due to different experimental settings (applied radioligand, whole cells *vs*. membranes, etc.), which emphasizes the importance of testing reference compounds for distinct experimental settings. Obtained K_i_ values for FE@SNAP and SNAP-7941 suggested intermediate affinity toward ADRB3 and thus potential ADRB3 mediated effects when pharmacological doses of unlabeled compounds are applied (e.g., *in vivo* blocking/displacement experiments). However, the affinity of both ligands toward MCHR1 is several thousand times higher than toward ADRB3. Due to the applied trace amounts of PET ligands (usually nanomolar range or lower), we conclude that [^18^F]FE@SNAP and [^11^C]SNAP-7941 are selective PET-tracers for MCHR1 imaging. Additionally performed real-time kinetic measurements using CHO-K1-ADRB3 cells and the yet rarely employed LigandTracer® technology provide a cell binding technique which is closer to *in vivo* imaging techniques like PET, as high resolution time-activity curves are generated by applying a single tracer concentration. Displacement of [^125^I]Iodocyanopindolol binding to CHO-K1-ADRB3 was achieved when concentrations ≥40 μM of unlabeled MCHR1 ligands were applied. This finding is in accordance with the K_i_ values in double digit μM-range obtained from competitive binding studies using cell membrane preparations and [^3^H]CGP-12177 as the respective radioligand.

Furthermore, whole cell binding experiments using mature brown adipocytes were performed to investigate the complexity of binding interactions apart from binding to a single receptor site, as in membrane preparations of CHO-K1-ADRB3 cells. Generally, experiments using whole cells account for unspecific processes including diffusion, nonspecific, and off-target binding—processes that are often overlooked when applying cell membranes. Thus, pre-adipocytes were cultivated and differentiated to mature brown adipocytes. Morphologic (histological staining of lipids) and metabolic ([^18^F]FDG uptake measurements) characteristics of the BAT phenotype were achieved 4–6 days after induction, thus, experiments using brown adipocytes were performed in this time frame. [^18^F]FDG uptake by mature brown adipocytes followed linear kinetics mirroring the unique trapping mechanism of [^18^F]FDG: it is transported *via* glucose transporters and intracellularly phosphorylated by hexokinase leading to trapping of the respective metabolite [^18^F]FDG-6-phosphate, as further metabolism is hindered (34–36). [^18^F]FDG uptake by undifferentiated pre-adipocytes was only minor indicating fibroblast-like functionality.

ADRB3 stimulation by the agonist CL 316,243 in brown adipocytes led to enhanced [^18^F]FDG uptake demonstrating brown adipocyte activation. This experiment served as the positive control, as this phenomenon is well described for brown adipose tissue in rodents (37, 38). Accordingly, the adrenergic beta receptor antagonist (S)-propranolol exerted no BAT activating effect. These results prove the expression of functional ADRB3 on the cultivated brown adipocytes used within this study. Interestingly, opposite effects (significant decrease in [^18^F]FDG uptake) are observed for pharmacological doses of SNAP-7941 and FE@SNAP. The role of MCHR1 in brown adipocytes, especially the involvement in glycolytic activity and glucose transporter expression is yet unexplored.

As accumulation of the MCHR1 PET-tracers was found in brown adipose tissue of naïve rats, specific binding to brown adipocytes was investigated *in vitro*. In competition binding experiments, the MCHR1 antagonist led to ~23% blocking of [^11^C]SNAP-7941 accumulation evincing specific binding to brown adipocytes. This finding points at MCHR1 expression in brown adipocytes and a subsequent involvement of the melanin-concentrating hormone system in brown fat depots. Western Blot analysis and qPCR finally showed the expression of the MCHR1 in murine brown adipocytes. In addition, as MCHR1 expression in lung is often discussed, we included mouse lung into our analysis. Our qPCR confirmed the abundance of *Mchr1* mRNA in mouse lung, which is in accordance with Kokkotou et al. (39). Furthermore, protein expression was detected in Western Blot analysis. Since neither qPCR nor Western Blot showed MCHR1 expression in spleen, we suggest it to be a legitimate negative control. This was further described by Kokkotou et al. (39). Although reference tissues were harvested from only two individuals, qPCR and Western Blot data are considered as representative. Further experiments are required to determine MCHR1 expression in rodent BAT with respect to age, sex, and diet.

In general, our findings support the initial theory of Ito et al. assuming that the anti-obesity effect of MCHR1 antagonists is not limited to a central action, but MCHR1 antagonists may also act peripherally in BAT (21). Expression of functional MCHR1 in rat white adipocytes has previously been demonstrated and a peripheral role for MCH in adipocytes in addition to its centrally mediated effects was already proposed by Bradley et al. (19, 40). Both, MCH and MCHR1 were found to be expressed in mouse and human pancreatic islets, thus, an autocrine role for MCH in the regulation of the hypothalamic-pancreatic axis was proposed (41). However, it was recently stated by Naufahu et al. that it is unknown whether MCH acts in a paracrine or autocrine manner or is released into the circulation (42). [^11^C]SNAP-7941 binding to brown adipocytes was blocked to a minor proportion by both the selective ADRB3 agonist CL 316,243 and the non-selective antagonist (S)-propranolol, demonstrating the superior affinity of [^11^C]SNAP-7941 toward MCHR1 compared to ADRB3. As (S)-propranolol displays even higher affinity for the adrenergic beta-1 and beta-2 receptor, [^11^C]SNAP-7941 binding to adrenergic beta receptor subtypes other than beta-3 can additionally be excluded (43). However, relatively high non-displaceable binding was found, which may be due to unspecific accumulation in lipids within brown adipocytes.

Moreover, these *in vitro* results are in accordance with the previously performed *in vivo* experiments administering [^18^F]FE@SNAP to conscious animals without the use of anesthesia, where significant blocking was achieved by a pharmacological dose SNAP-7941 (30). Contradictory results were obtained from μPET imaging, where binding enhancement of [^11^C]SNAP-7941 in BAT was shown after administration of a pharmacological dose of the unlabeled compound. PET imaging in small animals requires anesthesia in order to prevent laboratory animals from moving during the examination. However, anesthesia bears the disadvantage of possible molecular alterations, which may affect the examination's outcome, as it is already well-established for brain PET imaging (44). Effects of isoflurane, the used anesthetic agent within the mentioned *in vivo* study, on ADRB3 and/or MCHR1 expression are not described, though potential effects cannot be excluded.

Investigation of specific binding to brown adipocytes was solely performed for [^11^C]SNAP-7941, as extensive *in vitro* and *in vivo* investigations evinced the superior properties of [^11^C]SNAP-7941 compared to the fluoro-ethylated analog regarding radiochemical yield, metabolic stability, target affinity, selectivity, and imaging contrast (29). Thus, [^18^F]FE@SNAP was not further evaluated in this context.

The value of PET for BAT research was ultimately recognized when the radiolabeled glucose-analog, [^18^F]FDG was introduced for imaging and quantification of BAT activation. Although primarily perceived as a clinical tool, PET employing specific tracers can also add to basic research. In this way, MCHR1 PET imaging provided first evidence for MCHR1 expression in BAT and will contribute to the elucidation of the MCHR1 axis in BAT.

## Conclusion

The present study aimed at enhancing the understanding of the interplay between MCHR1 ligands and the pharmacologically complex brown adipose tissue. Uptake of [^11^C]SNAP-7941 and [^18^F]FE@SNAP in rat BAT gave the initial hint for MCHR1 expression in BAT. The herein performed *in vitro* binding studies, demonstrating specific [^11^C]SNAP-7941 binding to brown adipocytes and superior selectivity toward MCHR1 compared to ADRB3, provide first evidence for a direct involvement of the MCH/MCHR1 system in brown adipose tissue. Additionally, MCHR1 expression in murine brown adipocytes was demonstrated on mRNA and protein level for the first time.

## Ethics Statement

This study was carried out in accordance with the recommendations of the Institutional Animal Care and Use Committee of the Medical University of Vienna, Austria. The protocol was approved by the Austrian Ministry of Science, Research and Economy.

## Author Contributions

TB, CP, and MM conceived the study and designed the experiments. TB, KB, EK, and CP performed the experiments. CP performed the radiosyntheses of [^11^C]SNAP-7941 and [^18^F]FE@SNAP. OK and FK provided brown pre-adipocytes and established the induction protocol. GE designed the primers for qPCR. GE and LT helped with the establishment of the protocol for qPCR. KP and HS synthesized precursor compounds for radiolabeling and standard compounds. TB, CP, and EK analyzed the data and composed the draft. MM, GE, KW, WW, MH, and HV helped with outcome interpretation and reviewed the manuscript. All authors have given approval to the final version of the manuscript.

### Conflict of Interest Statement

Without relevance to this work, author WW is a part-time employee at the Center for Biomarker Research in Medicine–CBmed Ltd. The remaining authors declare that the research was conducted in the absence of any commercial or financial relationships that could be construed as a potential conflict of interest.
