# Supplementary material for: In vitro Radiopharmaceutical Evidence for MCHR1 Binding Sites in Murine Brown Adipocytes
Source: Front Endocrinol (Lausanne). 2019 Jun 11;10:324. doi: 10.3389/fendo.2019.00324 (PMC6581027; doi:10.3389/fendo.2019.00324)
Supplement: Supplementary file 1 [file Data_Sheet_1.docx]

Supplementary material

**1**

**2**

**3**

**4**


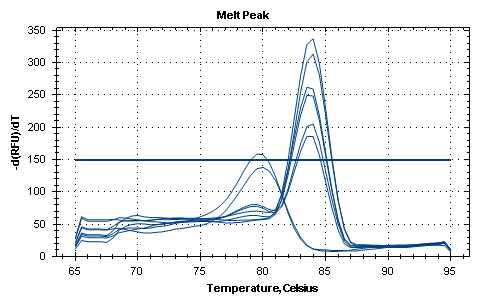


Representative melt curves of *Mchr1* primer in qPCR for mouse brain (1), murine brown adipocytes (2), mouse lung (3), and mouse spleen (4). Melt curves at ~ 84°C correspond to the dissociation of the specific product, whereas the curves at ~80°C represent the primer dimers.

Sequences of the primers:

mMCHR1_fo: AATGCCAGCAACATCTCCGA

mMCHR1_re: TGGATTTCTTCACCACGGCA

mActb_fo: GAAGATCTGGCACCACACCT

mActb_re: GTACATGGCTGGGGTGTTGA

mRpl27_fo: TACAACCACCTCATGCCCAC

mRpl27_re: AAACTTGACCTTGGCCTCCC
